# Supplementary material for: Two-dimensional metallic tantalum disulfide as a hydrogen evolution catalyst
Source: Nat Commun. 2017 Oct 16;8:958. doi: 10.1038/s41467-017-01089-z (PMC5643402; doi:10.1038/s41467-017-01089-z)
Supplement: Supplementary file 1 — Supplementary Information [file 41467_2017_1089_MOESM1_ESM.pdf]

Supplementary Note 1. X-ray photoemission spectroscopy spectrum of LPCVD-derived TaS_2 ultrathin films on Au foils

The X-ray photoelectron spectroscopy (XPS) measurement of LPCVD-synthesized TaS_2 ultrathin films on Au foils was firstly performed to determine the chemical composition, as well as the valence states of Ta and S (Supplementary Fig. 1). The spectrum of Ta 4f has two distinct peaks located at 22.7 and 24.7 eV, corresponding to the Ta $4f_{7/2}$ and $4f_{5/2}$ peaks, respectively, confirming that the Ta atoms in the TaS_2 film are in the Ta(+4) state[1].The S 2p spectrum shows two peaks at 162.1 eV and 163.2 eV, in line with S $2p_{3/2}$ and $2p_{1/2}$ peaks, revealing the −2 oxidation chemical state of S atoms in the TaS_2/Au sample.

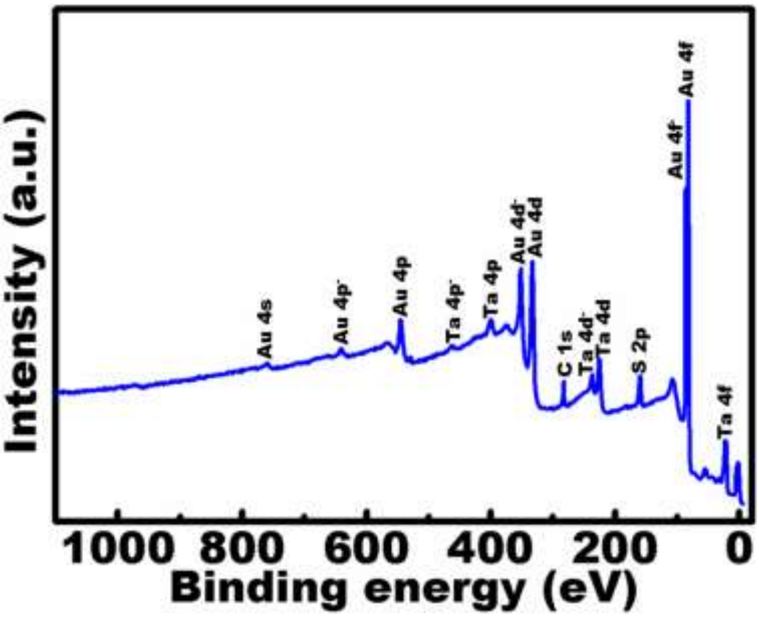

Supplementary Figure 1 | XPS spectrum of LPCVD-synthesized TaS_2 ultrathin films on Au foils acquired over a wide range of binding energies (0-1100 eV).

Supplementary Note 2. X-ray diffraction pattern of transferred TaS_2 ultrathin films on SiO_2/Si substrates

Supplementary Fig. 2 presents the X-ray diffraction (XRD) pattern of the transferred ultrathin TaS_2 film on SiO_2/Si (synthesized by LPCVD method with the growth time of ~20 min). It shows that, all the peaks of ultrathin TaS_2 films can be indexed as (00*l*) directions with the interlayer spacing of ~12.045 Å, which is closer to the lattice constant (*c*) of parent bulk 2H-TaS_2 (c = 12.097 Å), and larger than that of bulk 1T-TaS_2 (2c = 11.72 Å). The other (*hkl*) diffraction peaks typical for bulk 2H-TaS_2 were not observed in the TaS_2 ultrathin films, which possibly arise from the missing of the 2H symmetry. Such results indicate that, the LPCVD-synthesized TaS_2 ultrathin films possess 2H phase structure. This result is quite different from the previous report of the APCVD-synthesized 1T-TaS_2 on SiO_2/Si[2] at relatively low growth temperature (~750 ºC) and the use of slow cooling process, meanwhile, the different growth substrates.

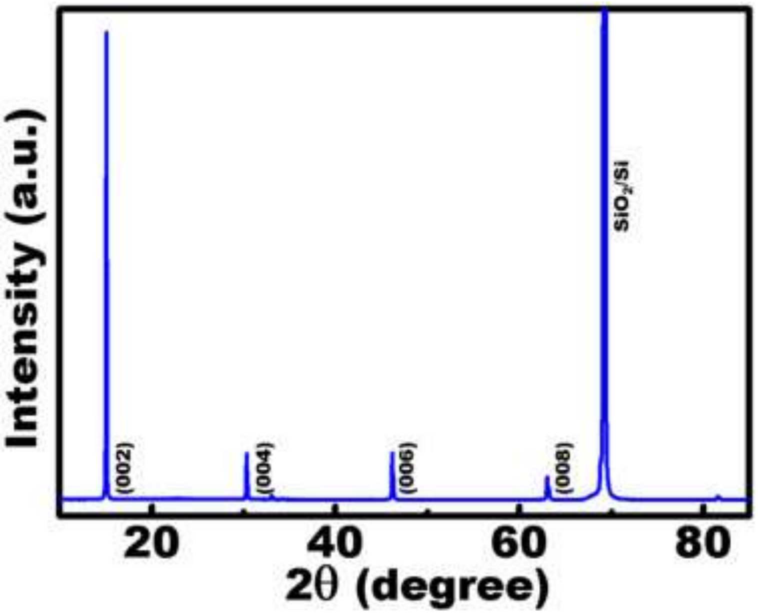

Supplementary Figure 2 | XRD pattern of transferred TaS_2 ultrathin films on SiO_2/Si showing its 2H phase feature.

Supplementary Note 3. Domain size and coverage evolution of LPCVD-derived 2H-TaS_2 with growth time

At the initial growth stage, triangular 2H-TaS_2 flakes (edge length of ~0.5 μm) were evolved and distributed uniformly on Au foils, as shown by the scanning electron microscope (SEM) image in Supplementary Fig. 3a. Atomic force microscopy (AFM) image and corresponding section-view analysis across the domain edge of the 2H-TaS_2 flake (after transferred on SiO_2/Si) show its apparent height of ~3 nm (inset of Supplementary Fig. 3a). Upon increasing the growth time to ~10 min (keeping other growth parameters identical), the edge length of 2H-TaS_2 triangles were enlarged to ~10 μm (Supplementary Fig. 3b). At ~20 min, full coverage 2H-TaS_2 films were obtained, as presented by the SEM image in Supplementary Fig. 3c. The thickness uniformity of 2H-TaS_2 domains and complete films were then convinced by the highly homogenous optical microscopy (OM) images (Supplementary Fig. 3d,e) in centimeter-size.

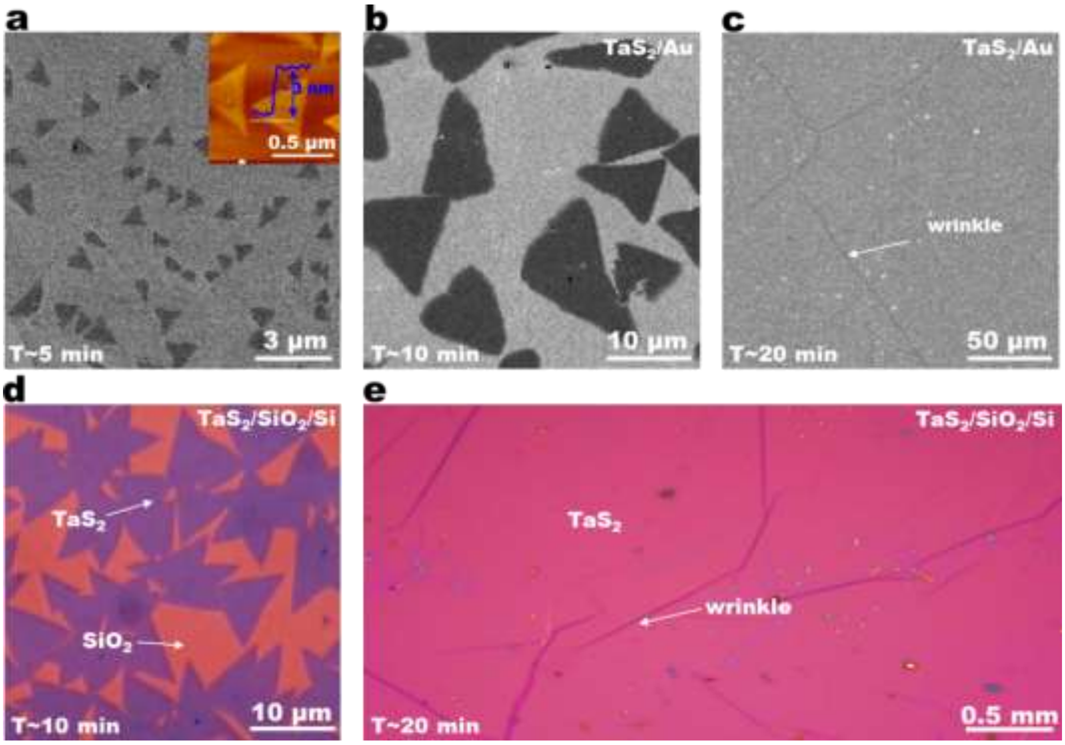

Supplementary Figure 3 | LPCVD synthesis of centimeter-size uniform 2H-TaS_2 films on Au foils. (**a-c**) SEM images of 2H-TaS_2 on Au foils with the growth time of ~5, ~10, and ~20 min, respectively (synthesized at 750 ºC under 100 sccm Ar and 10 sccm H_2 carrier gases). (**d**) OM image of transferred 2H-TaS_2 triangles on SiO_2/Si substrates (synthesized at 750 ºC for 10 min under 100 sccm Ar and 10 sccm H_2 carrier gases). (**e**) Large-area OM image showing the centimeter-size thickness uniformity of transferred 2H-TaS_2 films on SiO_2/Si (synthesized at 750 ºC for 20 min under 100 sccm Ar and 10 sccm H_2 carrier gases).

Supplementary Note 4. Growth mechanism illustration of 2H-TaS_2 on Au foils

As presented in Fig. 1g,j, the LPCVD-synthesized 2H-TaS_2 possess a rather uniform thickness of ~3 nm, which is totally different from the self-limited surface growth of monolayer MoS_2 or WS_2 on Au foils. In order to understand the growth mechanism of 2H-TaS_2 on Au foils, the growth time was further prolonged to ~30 min. Some 2H-TaS_2 flakes were evolved on the complete 2H-TaS_2 films, as shown in Supplementary Fig 4a. And such 2H-TaS_2 homojunction structures could be transferred onto arbitrary substrates (Supplementary Fig 4b). Interestingly, the thickness of the upper 2H-TaS_2 flake was also determined to be ~3 nm, as presented by the AFM image in Supplementary Fig. 4c. This result indicates that, there is a critical/magic thickness for the LPCVD-synthesized 2H-TaS_2 on Au foils, possibly due to the dimerization of Ta along the *c*-axis direction in a nanosized thickness.

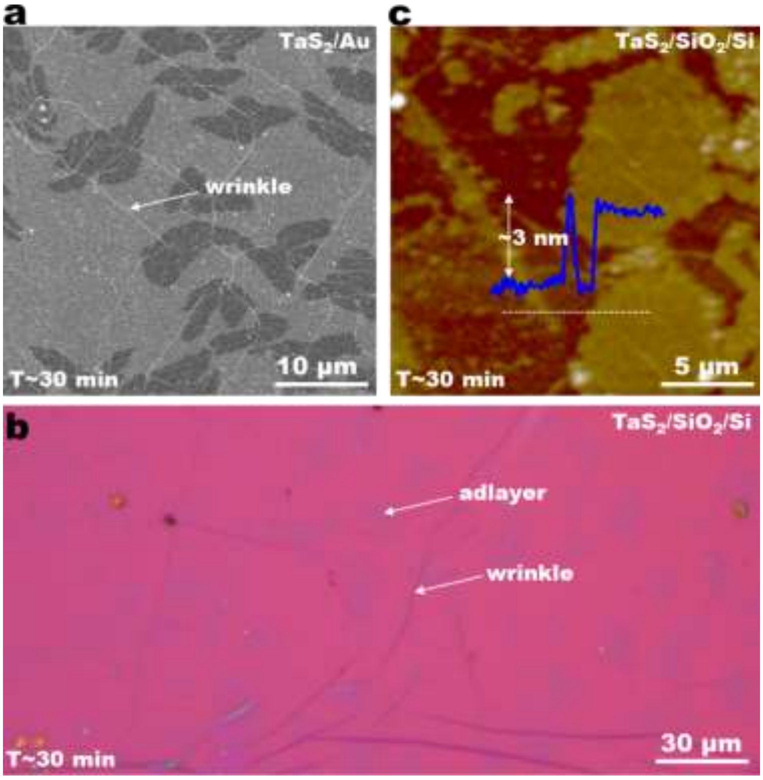

Supplementary Figure 4 | Growth mechanism illustration of LPCVD-derived 2H-TaS_2 on Au foils. (**a**) SEM image of 2H-TaS_2 on Au foils with the growth time of ~30 min (synthesized at 750 ºC under 100 sccm Ar and 10 sccm H_2 carrier gases). (**b**) OM image of transferred 2H-TaS_2 homojunction structures on SiO_2/Si (synthesized at 750 ºC for 30 min under 100 sccm Ar and 10 sccm H_2 carrier gases). (**c**) AFM image and height profile of transferred 2H-TaS_2 on SiO_2/Si showing a nominal thickness of ~3 nm for the upper 2H-TaS_2 flake.

Supplementary Note 5. APCVD synthesis of thickness-tunable 2H-TaS_2 flakes on Au foils

APCVD is an effective method to grow semiconducting TMDCs materials with tailored thicknesses[3,4], enabling to explore some novel physical issues associated with the dimensionality effect. Inspired by this, we selected an APCVD approach to realize the synthesis of 2H-TaS_2 directly on Au foils. The corresponding SEM images in Supplementary Fig. 5a,c,e,g reveal that, the edge length of hexagonal 2H-TaS_2 flakes are variable from ~5 to ~20 μm, upon increasing the growth time from ~3 to ~30 min. Notably, the thickness of such 2H-TaS_2 flakes are increased from ~15 to ~350 nm accordingly (Supplementary Fig. 5b,d,f,h). In this regard, the APCVD growth of 2H-TaS_2 on Au foils follows the Volmer-Weber (VW) growth mode, thus possessing wider tunability in layer thickness.

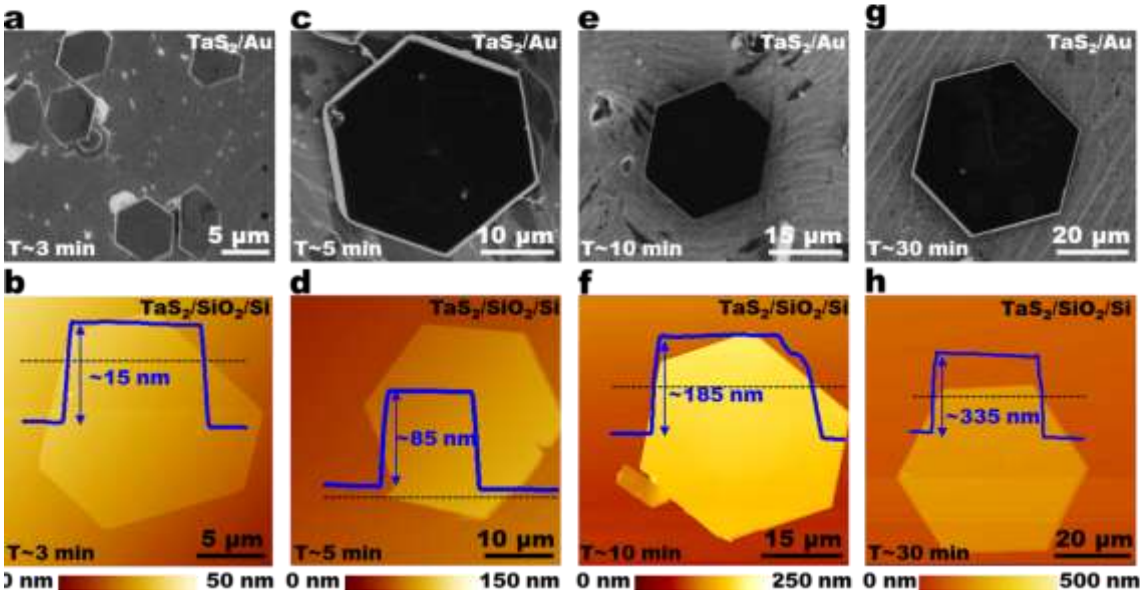

Supplementary Figure 5 | APCVD syntheses of thickness-tunable 2H-TaS_2 flakes on Au foils. (**a**,**c**,**e**,**g**) SEM images of 2H-TaS_2 flakes on Au foils with the growth time of ~3, ~5, ~10, and ~30 min, respectively (synthesized at 750 ºC under 100 sccm Ar and 10 sccm H_2 carrier gases). (**b**,**d**,**f**,**h**) Corresponding AFM images showing the tunable thicknesses of the transferred hexagonal 2H-TaS_2 flakes on SiO_2/Si.

Supplementary Note 6. X-ray photoemission spectroscopy spectrum of transferred TaS_2 flakes on SiO_2/Si (APCVD-derived sample)

The XPS measurement of transferred 2H-TaS_2 flakes on SiO_2/Si was also performed to exclude the possible mixing of Au in the 2H-TaS_2 layers during the CVD growth process (Supplementary Fig. 6). The Au 4f characteristic peaks are not observed, strongly indicating that Au is not penetrated into TaS_2 layers during the growth process.

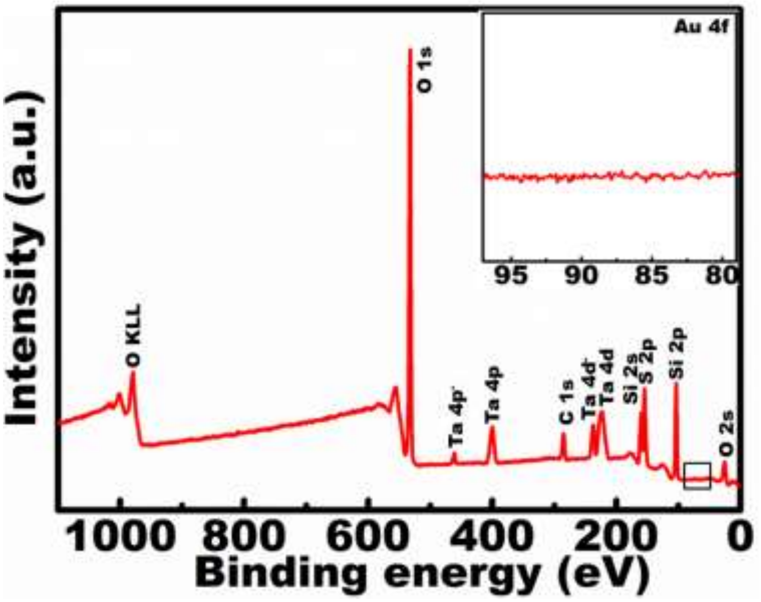

Supplementary Figure 6 | XPS spectrum of transferred 2H-TaS_2 on SiO_2/Si acquired over a wide range of binding energies (0-1100 eV). Inset is the zoon-in scan from the Au 4f range.

Supplementary Note 7. Transmission electron microscopy characterizations of LPCVD-synthesized 2H-TaS_2

The transmission electron microscopy (TEM) measurements were also performed on the transferred samples to determine the layer thickness, as well as the crystal structure of LPCVD-derived 2H-TaS_2. The high-resolution TEM (HRTEM) image captured from the domain edge of a 2H-TaS_2 flake stacking on a complete TaS_2 film possess a layer thickness of 8 (Supplementary Fig. 7a), namely, 4 plus 4. This data again confirms the existence of critical/magic thickness at the initial growth of 2H-TaS_2 films on Au foils through the LPCVD route. Moreover, the corresponding selected area electron diffraction (SAED) pattern in Supplementary Fig. 7b reveals only one set of hexagonally arranged diffraction spots, strongly suggestive the single-crystal feature of 2H-TaS_2. The Z-contrast spherical-aberration-corrected scanning transmission electron microscopy (STEM) image and the corresponding intensity line profile consistently show that, the atomic arrangement of TaS_2 obeys the 2H-phase atomic model with the Ta atoms octahedrally coordinated by S atoms (Supplementary Fig. 7c).

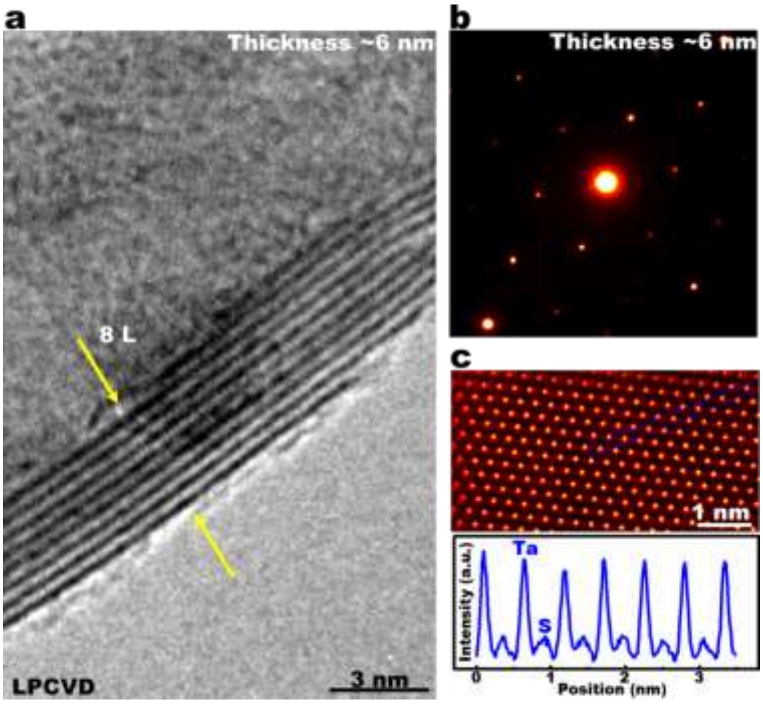

Supplementary Figure 7 | TEM characterizations of LPCVD-derived TaS_2 films. (**a**) HRTEM image along the domain edge of 2H-TaS_2 showing its 8-layer feature. (**b**) Corresponding SAED pattern captured within a 500 × 500 nm^2 area. (**c**) Atomic-resolution STEM-HAADF image revealing the perfect atomic lattices of TaS_2. The bottom panel is the corresponding intensity line profile along the blue rectangle.

Supplementary Note 8. Determine the charge-density wave phase transition temperature of 2H-TaS_2 by temperature-dependent Raman spectroscopy

Raman spectroscopy is an exquisitely sensitive and convenient technique to investigate both bulk and surface vibration modes of TMDCs[5,6], enabling to determine the charge-density wave (CDW) phase transition temperature of exfoliated 1T-TaS_2[7]. Supplementary Fig. 8a presents representative Raman spectra of 2H-TaS_2 (thickness of ~3 nm) upon cooling/warming processes. A negligible variation for the discernible peaks at different temperature can be noticed, suggesting that the CDW phase transitions of thinner TaS_2 layers are strongly suppressed, as similarly demonstrated by the electrical transport measurements of exfoliated 1T-TaS_2[8]. Intriguingly, as for the ~80-nm-thick 2H-TaS_2 flake, a broad Raman peak was clearly observed under 100 cm^{-1} at high temperature (>168 K) during the cooling process. In contrast, at low temperature (<168 K) and under such a range (under 100 cm^{-1}), some fine peaks appeared, indicating the appearance of NCCDW/CCDW phase transition with the transition temperature of ~168K. Notably, the same tendency was also recorded from the warming process but with the transition temperature upshifted to ~212 K (Supplementary Fig. 8b). The phenomenon was also observed for the ~150-nm-thick 2H-TaS_2 flake, with the transition temperature of ~150 and ~210 K for the cooling and warming processes, respectively (Supplementary Fig. 8c).

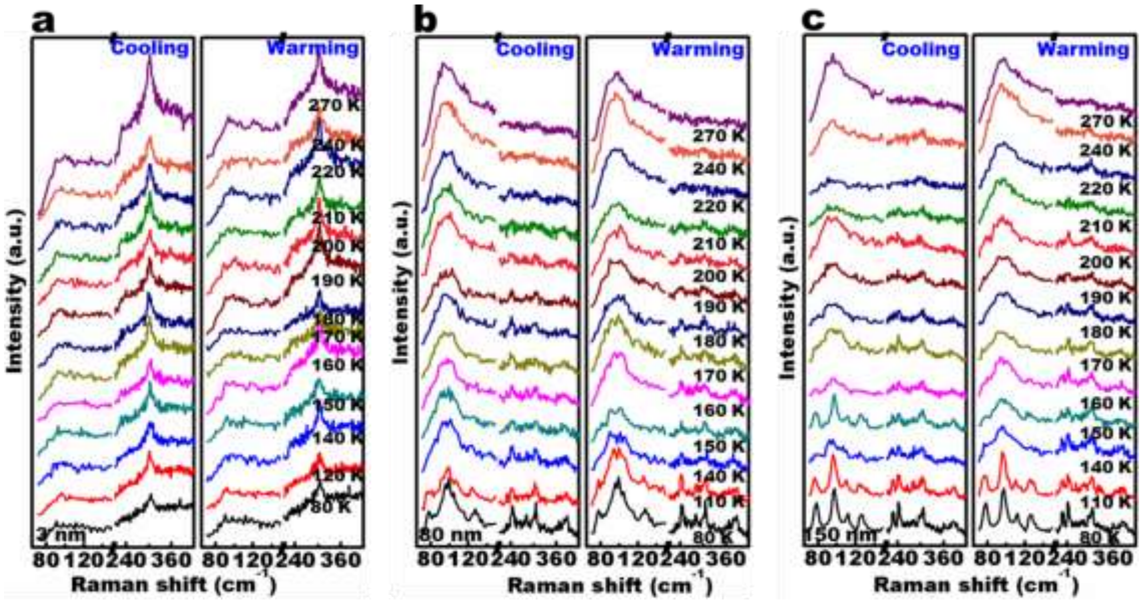

Supplementary Figure 8 | Temperature-dependent Raman characterizations of 2H-TaS_2 flakes with the thicknesses of ~3 nm (a), ~80 nm (b), and ~150 nm (c), respectively.

Supplementary Note 9. Determine the precise NCCDW/CCDW phase transition temperature of ~80-nm-thick 2H-TaS_2 flakes

The Raman frequencies of discernible peaks for ~80-nm-thick 2H-TaS_2 were plotted as a function of temperature upon cooling/warming processes in Supplementary Fig. 9a,b. The number of vibration modes and their frequencies were dramatically changed at the transition temperature T_c (marked by the dashed lines), and this temperature is different for the cooling ($T_{c,cool}$ = 168 K) and warming ($T_{c,warm}$ = 212 K) processes. The hysteresis temperature $\Delta T = T_{c,warm} - T_{c,cool}$ = 44 K and the average transition temperature $T_{c,avg} = (T_{c,warm} + T_{c,cool})/2$ = 190 K.

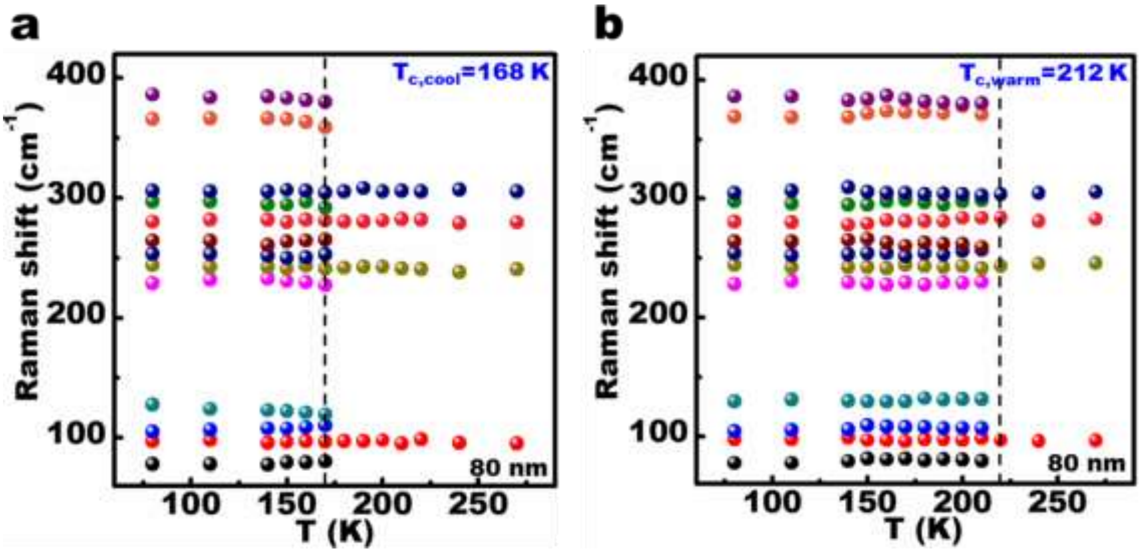

Supplementary Figure 9 | Raman frequency plots of discernible peaks for ~80-nm-thick 2H-TaS_2 flakes as a function of decreasing/increasing temperature, respectively.

Supplementary Note 10. Calculate the exchange current densities of 2H-TaS_2 with different thicknesses

By applying extrapolation method to the Tafel plots, we have calculated the exchange current densities (j_0) of 2H-TaS_2 with different thicknesses as shown in Supplementary Fig. 10. A remarkable j_0 of ~179.47 µA/cm^2 can be noticed which is by far the highest value among the reported TMDCs-based catalysts[9–11].

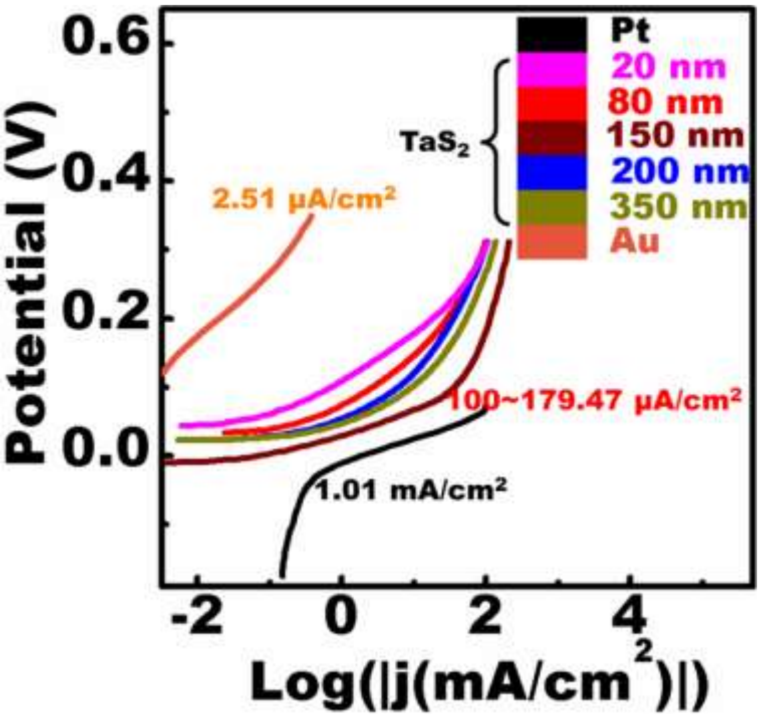

Supplementary Figure 10 | Calculated exchange current densities of 2H-TaS_2 flakes with different thicknesses by applying an extrapolation method to the Tafel plots.

Supplementary Note 11. The polarization curves of 2H-TaS_2 (with different thicknesses) at the initial and after electrochemical cycles

The polarization curves of 2H-TaS_2 (with different thicknesses) recorded at the initial and after electrochemical cycles convince the enhanced electrocatalytic activity (Supplementary Fig. 11).

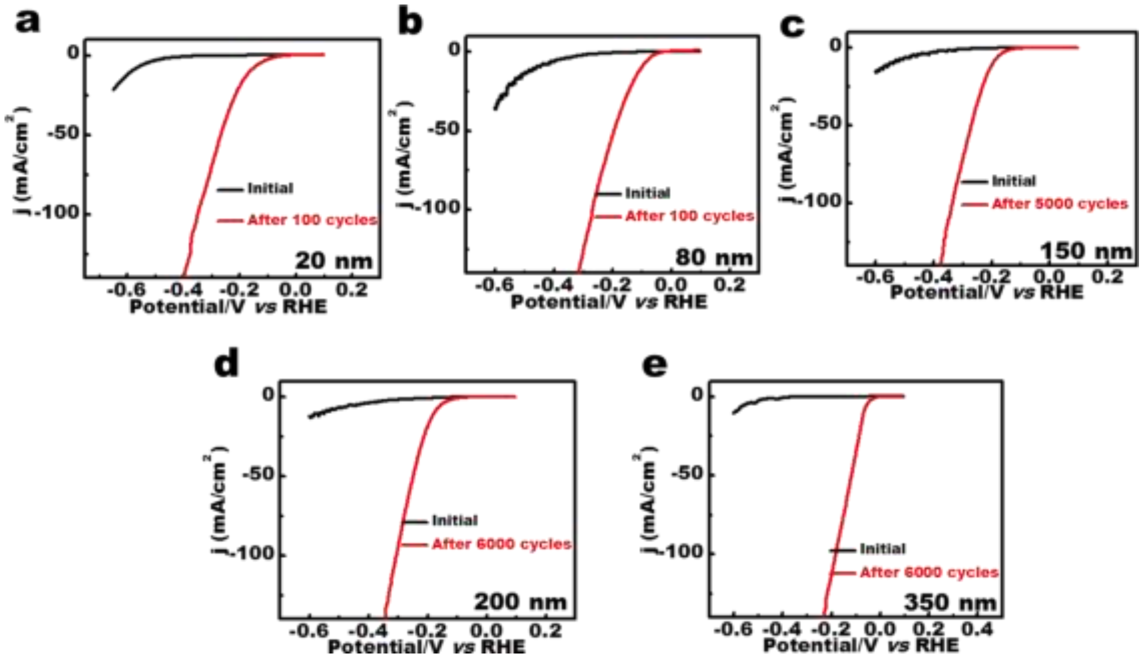

Supplementary Figure 11 | Polarization curves (*i*R-corrected) of 2H-TaS_2 with different thicknesses through different electrochemical cycles.

Supplementary Note 12. The morphology evolution of 2H-TaS_2 flakes at the initial and after electrochemical cycles

The morphology of as-grown 2H-TaS_2 flake on Au foils is shown by the SEM image in Supplementary Fig. 12a. Interestingly, after 5000 electrochemical cycles, the 2H-TaS_2 become thinner, smaller, and more disperse (Supplementary Fig. 12b). The self-optimizing morphological change of 2H-TaS_2 domains is proposed to enhance the electrocatalytic HER performance.

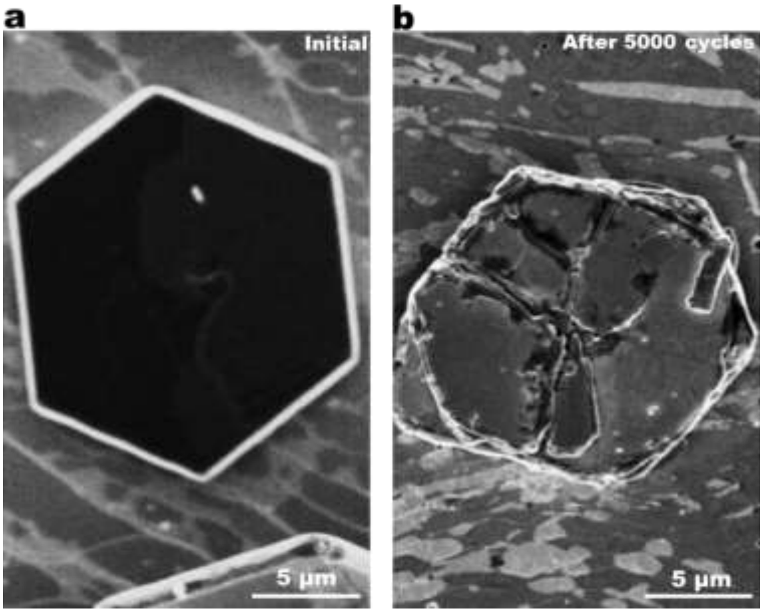

Supplementary Figure 12 | SEM images of 2H-TaS_2 flakes on Au foils before (a) and after (b) 5000 electrochemical cycles.

Supplementary Note 13. Chemical composition analysis of 2H-TaS_2 before and after electrochemical cycles

The XPS characterizations of 2H-TaS_2 samples before and after HER measurements are then performed and displayed in Supplementary Fig. 13. Notably, the Pt characteristic peaks are not observed in the XPS data. This result indicates that Pt is not covered on or mixed with TaS_2 during the HER process (Supplementary Fig. 13a). Interestingly, there is negligible variation for Ta 4f and S 2p signals (Supplementary Fig. 13b,c), highly suggestive the invariable chemical composition of 2H-TaS_2 throughout the electrocatalytic processes.

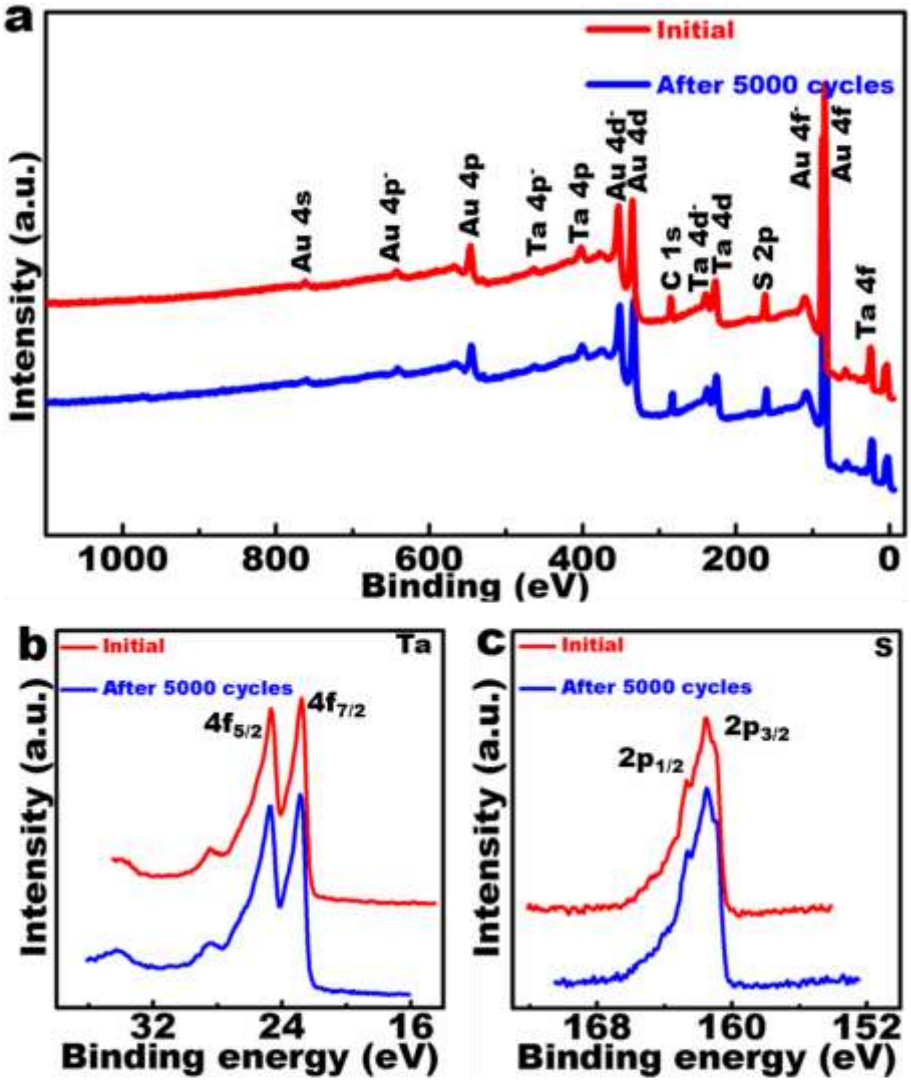

Supplementary Figure 13 | (**a**) XPS over a wide range of binding energies (0-1100 eV) Ta (**b**) and S (**c**) signals of 2H-TaS_2 on Au foils before and after 5000 electrochemical cycles, convincing the stability of 2H-TaS_2 during the electrocatalytic processes.

Supplementary Note 14. HER performance of 2H-TaS_2/Au by using carbon rod as the counter electrode and by using the Nafion proton exchange membrane assisted electrochemical measurements

In order to rule out the possible effect of Pt contamination, we have re-measured the HER performance of various 2H-TaS_2 samples on Au foils by using the carbon rod as the counter electrode. The overpotentials at the cathodic current density of 10 mA/cm^2 are falling in the range of 100–190 mV, and the Tafel slope values are in the range of 50–55 mV/dec. Such results approach to those obtained from the Pt counter electrode based HER measurements. Additionally, by applying extrapolation method to the Tafel plots, the exchange current densities (j_0) are also achieved in the range of 85–143.2 μA/cm^2, similar with those of using the Pt counter electrode (Supplementary Fig. 14a–c, and Supplementary Table 1). Notably, such three parameters are very close to the results derived by using Pt as the counter electrode. Briefly, the Pt counter electrode has very small effect on the HER performance of 2H-TaS_2. And the slightly larger Tafel slope value than that of using the Pt counter electrode based HER measurement may be induced by the relatively low conductivity of the carbon rod. We also performed the Nafion proton exchange membrane assisted electrochemical measurements by using Pt counter electrode. This is because the proton exchange membrane only permits the transfer of protons but impedes the other species. The overpotentials for the different samples at j = 10 mA/cm^2 are in the range of 80–160 mV, the Tafel slopes are 45–49 mV/dec, and the j_0 values are 90–149.5 μA/cm^2, such values are very close to the results only by using Pt as the counter electrode (Supplementary Fig. 14d–f, and Supplementary Table 1). This result again suggests the negligible effect of Pt contamination on the HER performance of TaS_2.

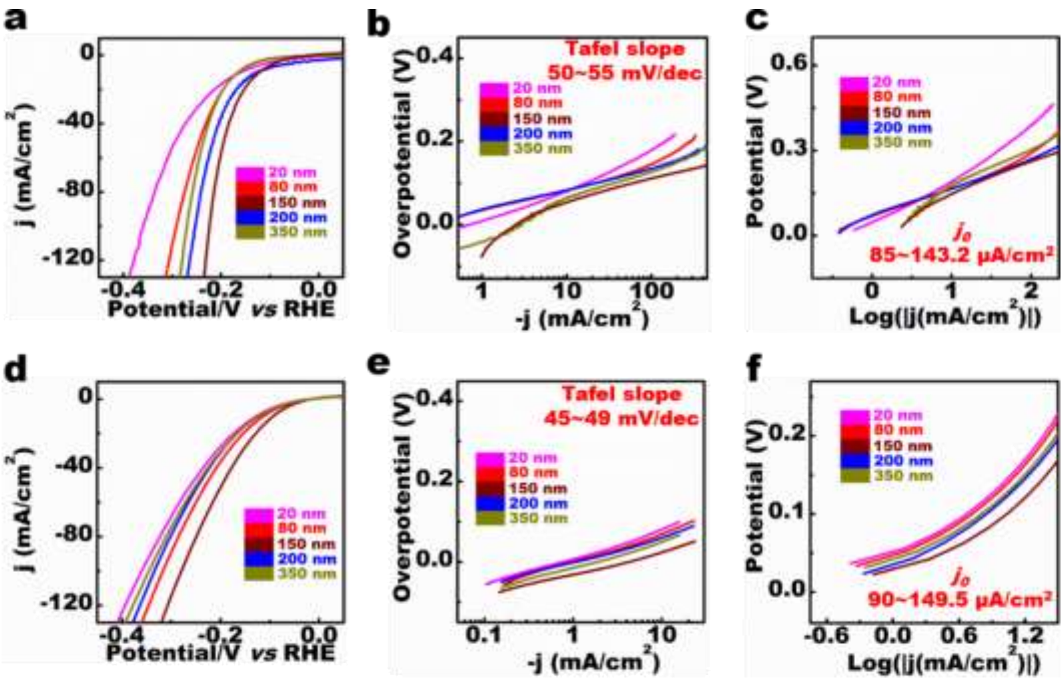

Supplementary Figure 14 | Additional HER measurements of as-grown 2H-TaS_2 by using carbon rod as the counter electrode and by using the Nafion proton exchange membrane assisted electrochemical measurements (with Pt counter electrode). (**a**–**c**) Polarization curves (*iR*-corrected), corresponding Tafel plots, and calculated exchange current densities of as-grown 2H-TaS_2 with different thicknesses by using carbon rod as the counter electrode. (**d**–**f**) Polarization curves (*iR*-corrected), corresponding Tafel plots, and calculated exchange current densities of as-grown 2H-TaS_2 with different thicknesses achieved by Nafion proton exchange membrane assisted electrochemical measurements.

Supplementary Note 15. Electrochemical impedance spectra (ESI) of 2H-TaS_2 at the initial and after electrochemical cycles

The electrochemical impedance spectra (ESI) of 2H-TaS_2 with different thicknesses were shown in Supplementary Fig. 15. Decreased charge-transfer resistances were clearly observed upon cycling. Such results convince the shortened interlayer electron-transfer pathways for the thinner 2H-TaS_2 after electrochemical cycles.

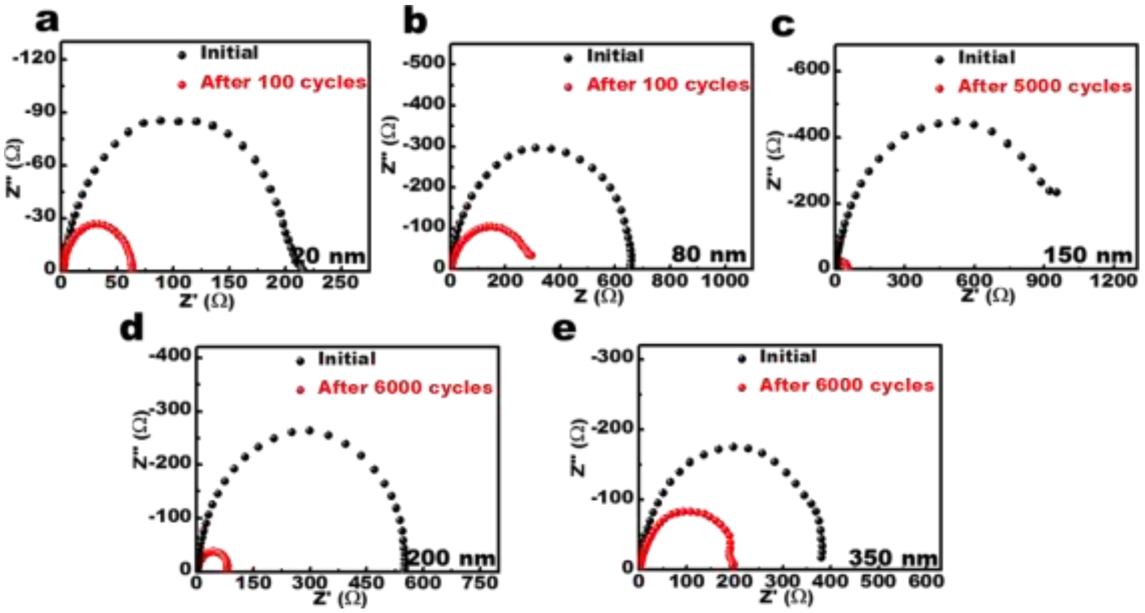

Supplementary Figure 15 | ESI of 2H-TaS_2 flakes with different thicknesses before and after electrochemical cycles.

Supplementary Note 16. HER performances of TaS_2 synthesized at different temperature

The HER performances of TaS_2 samples synthesized at different temperatures were also measured to detect the effect of synthesis temperature on the catalytic activity (Supplementary Fig. 16a,b). In order to exclude the influence of the edge density difference on the hydrogen evolution rate, the TaS_2 samples were selected to possess the similar coverage (~70%) and domain size (edge length of ~5 μm). Notably, the HER performances of TaS_2 synthesized at 650, 700, and 750 ºC are very similar, featured with the overpotential values (at the cathodic current density (*j*) of 10 mA/cm^2) of 120–125 mV, and the Tafel slope values of 45–48 mV/dec. This phenomenon indicates that, the growth temperature (from 650 to 750 ºC) has negligible effect on the HER performance of TaS_2.

However, for the TaS_2 flakes synthesized at high temperature (800 and 850 ºC), the overpotential (j = 10 mA/cm^2) and Tafel slope values are raised to 152–171 mV and 58–62 mV/dec, respectively. Liu, Z., and Yakobson, B. I. *et al*. have demonstrated that, 1T-TaS_2 is dominant for the high temperature (900 ºC) synthesized sample[2,12]. Corresponding DFT calculation reveals that, 1T-TaS_2 has relatively low catalytic activity[13]. Herein, we speculate that the high temperature (800 and 850 ºC) synthesized samples contain 1T-TaS_2, resulting in their reduced HER performance. For more proof, the Raman spectra of transferred TaS_2 (synthesized at 800 and 850 ºC) on SiO_2/Si are also collected to determine the phase state, as shown in Supplementary Fig. 16c,d. Notably, the Raman characteristic peaks of 2H-TaS_2 (E_{2g} ~286.4 cm^{-1} and A_{1g} ~401.4 cm^{-1}) and 1T-TaS_2 (E_{2g} ~244.2 cm^{-1} and A_{1g} ~396.5 cm^{-1}) are observed concurrently, confirming that 1T- and 2H-TaS_2 are coexisting for the high temperature (800 and 850 ºC) synthesized samples. Briefly, the low synthesis temperature (<800 ºC) has very little effect on the HER performance of the CVD-derived TaS_2, and the high synthesis temperature (>800 ºC) reduces the catalytic activity of TaS_2. This can be explained from the generation of different phases.

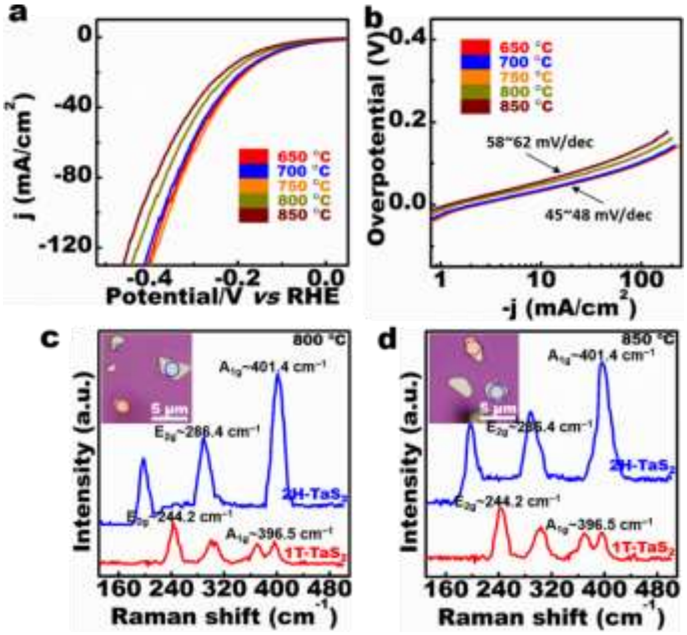

Supplementary Figure 16 | HER measurements of TaS_2 synthesized at different temperature (650, 700, 750, 800, and 850 ºC). (**a**,**b**) Polarization curves (*iR*-corrected) and corresponding Tafel plots of TaS_2 synthesized at different temperature (650, 700, 750, 800, and 850 ºC). (**c**,**d**) Raman spectra of TaS_2 synthesized at different temperature (800 and 850 ºC).

Supplementary Table 1. Comparison of the HER performances of 2H-TaS_2 by using different test methods

Method	Overpotential at 10 mA/cm^2 (mV *vs* RHE)	Tafel slope (mV/dec)	Exchange current density ($\mu A/cm^2$)
Pt counter electrode	65–150	33–42	100–179.47
carbon rod counter electrode	100–190	50–55	85–143.2
Nafion proton exchange membrane	80–160	45–49	90–149.5

Supplementary References

1. Li, H. *et al*. Atomic-sized pores enhanced electrocatalysis of TaS_2 nanosheets for hydrogen evolution. *Adv. Mater.* **28,** 8945–8949 (2016).
2. Fu, W. *et al*. Controlled synthesis of atomically thin 1T-TaS_2 for tunable charge density wave phase transitions. *Chem. Mater.* **28,** 7613–7618 (2016).
3. Ling, X. *et al*. Role of the seeding promoter in MoS_2 growth by chemical vapor deposition. *Nano Lett.* **4,** 464–472 (2014).
4. Gurarslan, A. *et al*. Surface-energy-assisted perfect transfer of centimeter-scale monolayer and few-layer MoS_2 films onto arbitrary substrates. *ACS Nano* **8,** 11522–11528 (2014).
5. Lee, Y. *et al.* Synthesis of large-area MoS_2 atomic layers with chemical vapor deposition. *Adv. Mater.* **24,** 2320–2325 (2012).
6. Shi, Y. M. *et al*. van der Waals epitaxy of MoS_2 layers using graphene as growth templates. *Nano Lett.* **12,** 2784–2791 (2012).
7. He, R. *et al*. Distinct surface and bulk charge density waves in ultrathin 1T-TaS_2. *Phys. Rev. B* **94,** 201108 (R) (2016).
8. Yu, Y. J. *et al*. Gate-tunable phase transitions in thin flakes of 1T-TaS_2. *Nat. Nanotechnol.* **10,** 270–276 (2015).
9. Jaramillo, T. F. *et al*. Identification of active edge sites for electrochemical H_2 evolution from MoS_2 nanocatalysts. *Science* **317,** 100–102 (2007).
10. Voiry, D. *et al*. The role of electronic coupling between substrate and 2D MoS_2 nanosheets in electrocatalytic production of hydrogen. *Nat. Mater.* **15,** 1003–1009 (2016).
11. Yu, Y. F. *et al*. Layer-dependent electrocatalysis of MoS_2 for hydrogen evolution. *Nano Lett.* **14,** 553–558 (2014).
12. Liu, Y. Y. *et al*. Self-optimizing layered hydrogen evolution catalyst with high basal-plane activity. Preprint at https://arxiv.org/abs/1608.05755 (2016).
13. Tsai, C., Chan, K., Nørskov, J. K. & Abild-Pedersen, F. Theoretical insights into the hydrogen evolution activity of layered transition metal dichalcogenides. *Surf. Sci.* **640,** 133–140 (2015).
